# How to increase COVID-19 vaccination among a population with persistently suboptimal vaccine uptake? Evidence from the North Macedonia mobile vaccination and public health advice caravan

**DOI:** 10.1016/j.healthpol.2023.104966

**Published:** 2024-01

**Authors:** Manuel Serrano-Alarcón, Martin Mckee, Leonardo Palumbo, Cristiana Salvi, Anne Johansen, David Stuckler

**Affiliations:** a European Commission, Joint Research Centre (JRC), Ispra, Italy; b Dondena Centre for Research on Social Dynamics and Public Policy, Bocconi University, Italy; c Department of Health Services Research and Policy, London School of Hygiene and Tropical Medicine, University of London, London, United Kingdom; d World Health Organization, Geneva, Switzerland; e Department of Social & Political Sciences, Bocconi University, Milano, Italy

**Keywords:** Vaccination, Covid-19, Community-based intervention, Vaccine hesitancy

## Abstract

Over three years since the first COVID-19 vaccine was approved, many countries still have suboptimal vaccination rates despite holding great amounts of vaccines. Overall, there is little evidence on which policies are more effective to encourage vaccination, particularly in countries where a large share of the population remains unvaccinated. In this study, we examine the effectiveness of a community-based intervention carried out in March 2022 in North Macedonia, a country with a large and persistent share of the population that remains unvaccinated. The intervention, spearheaded by the Ministry of Health and supported by the World Health Organization and UNICEF, consisted of a mobile caravan offering vaccination and public health advice to different locations across the country on different days. Results from our staggered difference-in-difference model show that the mobile vaccination caravan increased daily vaccination rates by 7.7 vaccines per 100,000 inhabitants during the three weeks after the day of the caravan visit. This corresponds to a 35 % increase with respect to pre-intervention vaccination rates. We estimate a cost-effectiveness of 25.4 US dollars (USD) per additional vaccination induced. These results point to mobile caravan vaccines as an effective and cost-effective strategy to increase COVID-19 vaccination rates, even in a context of persistently suboptimal uptake.

## Highlights

• We examine the effectiveness of a COVID-19 vaccination caravan in North Macedonia.
• The caravan increased vaccinations by 35 % during 3 weeks after the visit.
• We estimate a cost-effectiveness of 25.4 US dollars per additional vaccination induced.
• Mobile vaccination caravans can be cost-effective interventions to increase COVID-19 vaccination rates.

## 1 Introduction

Over three years since the first COVID-19 vaccine was approved, many countries still have sub-optimal vaccination rates despite holding large amounts of vaccines. Some are even destroying expired vaccine stocks because of low demand [1]. They have sought to increase vaccine uptake using a range of measures. Broadly categorized, some seek to compel or encourage uptake, for example with vaccine mandates or monetary incentives, respectively. Others aim to increase knowledge through public health advice campaigns or overcome barriers by enhancing the accessibility of vaccination facilities [2]. However, there is still little evidence about whether and to what extent these policies are effective, particularly in countries where a large share of the population remains unvaccinated.

There is some evidence from randomized control trials on the effectiveness of targeted interventions. Small monetary incentives in Sweden [3] have been found effective as have nudges such as reminders and public health messages in a range of European countries, New Zealand and the US [4,5]. Government-mandates on vaccination certificates have been found to increase uptake by, typically, 5 to 10 percentage points in high-income countries with high vaccination rates [6]. However, these mandates may also generate a political backlash and raise ethical objections from those who see them as restricting personal freedoms [7]. Vaccine lotteries, in which those agreeing to be vaccinated have a chance of winning a reward, have had more modest results in those US states that have implemented them [8,9]. Finally, mobile vaccination units have been also implemented across the US and Europe with the objective of bringing vaccination services closer to the target population [10], [11], [12]. Mobile vaccination units have been shown to increase vaccination rates in the UK by 23 % [13] and in Switzerland by more than threefold [14]. The authors suggest that their results may be attributed to several factors. These include easier access to vaccination [13], reduced administrative barriers [14], or the enthusiasm generated by a “vaccination event” [14].

However, a major limitation of these prior studies is that they focused on high-income countries, with high vaccination rates and at an early stage of the vaccination process [8,[3], [4], [5],^14^,^9^,^13^]. It is thus uncertain how effective these programs would be where a majority of the population has persistently sub-optimal levels of vaccine uptake.

This scenario is particularly relevant in most low- and middle-income countries (LMICs) where COVID-19 vaccine uptake has remained sub-optimal [15]. Community-based interventions have previously played an important role in outbreak control in LMICs, with recent examples including mpox and Ebola. These interventions may include community surveillance, community education and community vaccination programs, among others [16]. Drawing from these experiences, community-based interventions have emerged as a means to boost COVID-19 vaccination in LMICs. As an example, an initiative employing community champions (including people previously vaccinated and local leaders) significantly increased COVID-19 vaccination rates in low-uptake regions of Tanzania, rising from 10 % to 94 % [17]. However, generalising from such studies is difficult given the importance of context. A community-based intervention may operate differently depending on factors such as geography, healthcare system organisation, political institutions, social norms, etc.

The closest study to ours in LMICs is the one conducted by Abdullah et al. [18] in Pakistan. They evaluated the effectiveness of an intervention involving mobile vaccination units and an awareness campaign led by community leaders. This achieved a 17 % improvement in vaccination rates, although confined to only one of three treated areas. In the remaining areas, increased willingness to get vaccinated did not translate into higher uptake. The researchers attributed this to limited access in remote settlements, with increased vaccination units insufficient to overcome this issue.

In our study, we examine the effectiveness of a community-based intervention, led by the Ministry of Health and with support from the 10.13039/100004423World Health Organization at regional, hub and country levels and UNICEF, carried out in North Macedonia in March-April 2022, a country with a sub-optimal uptake of COVID-19 vaccination; only 45 % of the population had been vaccinated by the beginning of the intervention.^1^ The intervention involved sending a mobile vaccination and public health advice caravan to different locations.

Our study provides evidence on the effectiveness of a community-based intervention in an understudied context, North Macedonia, an upper middle-income country with sub-optimal vaccine uptake. This situation mirrors that of most of its neighbouring countries of the Balkan region [19,20]. Consequently, our findings can provide valuable insights for the development of vaccination policies within this region.

## 2 COVID-19 vaccination in North Macedonia and the caravan

**Fig. 1:**
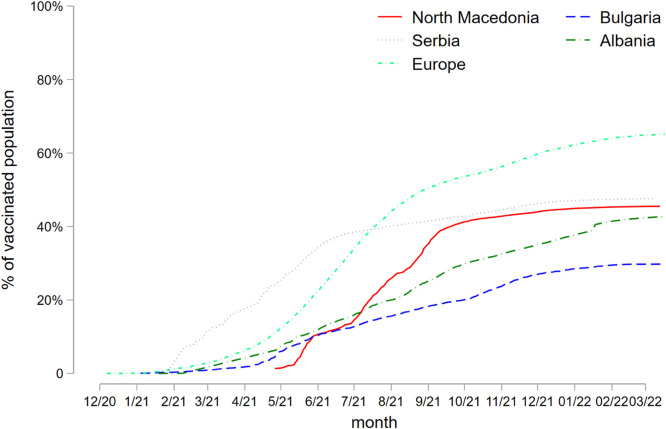
Fully vaccinated population in North Macedonia and selected countries **NOTES:** Europe region includes the following countries: Albania, Andorra, Austria, Belarus, Belgium, Bosnia and Herzegovina, Bulgaria, Croatia, Cyprus, Czech Republic, Denmark, Estonia, Finland, France, Germany, Greece, Hungary, Iceland, Ireland, Italy, Kosovo, Latvia, Liechtenstein, Lithuania, Luxembourg, Malta, Moldova, Monaco, Montenegro, Netherlands, North Macedonia, Norway, Poland, Portugal, Romania, Russia, San Marino, Serbia, Slovakia, Slovenia, Spain, Sweden, Switzerland, Ukraine, United Kingdom. We have extracted the data on the percentage of the population fully vaccinated by country directly from “Our world in data” database [28]. For North Macedonia however, we only extracted the total number of individuals fully vaccinated and divided this by the updated total population from the 2021 North Macedonia Population Census 2021 (i.e. 1836,713) to calculate the percentage of the population fully vaccinated. Note that “Our world in data” uses instead population data from the old 2002 census (i.e. over 2 million people) to calculate the percentage of population vaccinated for North Macedonia.

North Macedonia is one of the countries with the lowest COVID-19 vaccination rates in Europe. By the beginning of the intervention, in March 2022, around 45 % of the population was fully vaccinated (i.e.: had received two doses of the vaccine). This is similar to the situation in most of the Balkan region (Fig. 1), where countries report vaccination rates between 20 and 30 percentage points lower than the average in Europe (64 % by March 2022). Some scholars have explained this as a consequence of a high level of distrust in national governments, the pervasive presence of the anti-vaccine movement, and widespread misinformation on social media [19,20]. A survey among North Macedonian healthcare professionals pointed to other country-specific factors such as the failure of the institutions to communicate the benefits of vaccination, particularly to those with comorbidities who fear side effects, and communication of vaccine hesitancy from vaccine-hesitant healthcare professionals to patients [21].

The COVID-19 caravan was a community-based intervention carried out in partnership between the World Health Organization, United Nations International Children´s Emergency Fund (UNICEF), the United States Agency for International Development (USAID), and North Macedonia authorities. It involved sending a mobile vaccination unit to areas with sub-optimal COVID-19 vaccination uptake to increase vaccination rates. Healthcare workers accompanied the mobile vaccination unit (the caravan) disseminating public health advice on COVID-19 protective measures (e.g. giving out brochures and promotional material, one-to-one conversations with patients and local healthcare workers, engaging with local media, etc.). The caravan visited one of 14 locations each day from the 21st of March to the 3rd of April of 2022. From 80 municipalities, 22 were exposed to the 14 caravan visits. Importantly, one of these 14 locations was the main shopping centre of the capital, Skopje, in the municipality of Gazi Baba (Skopje). Skopje has a total of 10 municipalities. This shopping centre is visited by citizens from all the municipalities within Skopje. We then assigned as treated municipalities as of the day of the shopping centre visit the remaining 8 municipalities within Skopje that had not had any caravan visit before. As a result, we have 22 treated municipalities over the 14 days of caravan visits (See Table A1 in Appendix A for a list of these municipalities and Appendix B for more details about the caravan).

## 3 Methods

### 3.1 Data

We use data on daily vaccines administered per municipality for the 80 municipalities of North Macedonia during the period from 1st March to 17th April. We include any vaccination administered in the municipality, either at the caravan or in any healthcare facility, given our need to capture any lagged effects attributable to the vaccine promotion activities.^2^ We then observe each of the treated (i.e. visited by the caravan) municipalities up to an average of three weeks after the COVID-19 caravan visit.^3^ WHO Europe and its partners provided data on the number of vaccines administered per municipality per day, and municipality population (based on North Macedonia Population Census 2021). From this, we calculate vaccine rates as the daily number of administered vaccines per 100,000 inhabitants per municipality. We also analyse data on the cost of the intervention from both the vaccine promotion activities and the administration of the vaccine itself.

### 3.2 Empirical strategy

We exploit the staggered implementation of the COVID-19 vaccine caravan by comparing the evolution of vaccine rates among those municipalities that received a visit of the caravan vs those that did not in a series of difference-in-difference (DiD) models. In total, 22 municipalities received the caravan and 58 did not. The outcome variables of all our models are daily vaccine rates per 100,000 inhabitants measured at the municipality level. All the details (including equations) of the DiD models are explained in Appendix C.

First, we utilize a DiD model in an event study-like specification to study the effect of the intervention relative to the time of the caravan visit (See model 1 of Appendix C for a full specification of the model). In this model we test for the presence of pre-treatment parallel trends, a key assumption in the difference-in-difference model as it is necessary to show that there were no differences in trends in treated and control municipalities before the intervention. For this to be true the coefficients prior to the intervention should not be statistically significant from zero. Coefficients after the day of the intervention measure the effect of the caravan each day after the intervention, up to 3 weeks after the intervention. In this model 1 all estimates are estimated with respect to the day prior to the intervention (which serves as the baseline in the model).

Second, to study the dynamics of the effect over time further and avoid setting an arbitrary day as a baseline for our estimates, we estimate another DiD model (see model 2 in Appendix C) measuring the effect with respect to the full period before the caravan visit, not only with respect to the day before (as it was in model 1).^4^

Finally, we calculate the average treatment effect for the full post-treatment period with a two-way fixed effects (TWFE) model (see model 3 in Appendix C). This model measures the average treatment effect on daily vaccine rates during the three weeks after the intervention. Recent literature has shown how analysing a staggered intervention with a canonical TWFE model may be biased due to the use of early treated units as controls [22,23]. We perform the Goodman-Bacon decomposition to assess which comparisons have the most weight in our estimates and show that only 2.3 % of our comparisons used early treated municipalities as controls (Table A2 in Appendix A). This suggests that the canonical two-way fixed effects is adequate in our context. Still, in the robustness check in Section 3.3 we also carry out the Sant'Anna and Zhao [24] estimator, which uses only not-yet treated municipalities as controls and our results remain unchanged

## 4 Results

### 4.1 Descriptive statistics

Since the intervention was not randomly implemented, there are some differences between treated and control municipalities (Table A3 in Appendix A). Treated municipalities have a larger and younger population and a higher share of the population are foreign-born. In contrast, they have similar levels of education and unemployment. Still, it is important to note that the main assumption under the difference-in-difference design is not that treated and control units must be similar in all characteristics but rather that they should follow a parallel trend in the outcome variable in the absence of the treatment, and therefore the trend in vaccine rates should not be related with the caravan visit. We formally test for this assumption in Section 4.2. Model 1 finds no significant coefficients before the intervention, which indicates that trends in vaccination rates were similar in treated and control municipalities at that time.

**Fig. 2:**
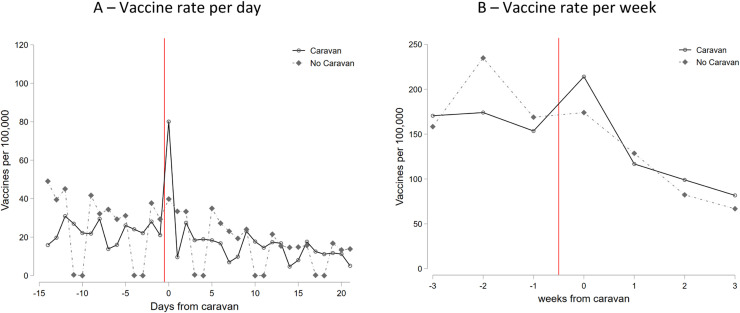
Vaccine rates over time normalizing to 0 the day (or week) of the caravan visit NOTES: For the treated group, we normalized to zero the day (or week) of the caravan visit in the x-axis. For the control group, in Figure A we set equal to zero the 23th of March, the Wednesday of the first week of the intervention; in Figure B we set equal to zero the week from 21th to 27th of March.

Before the intervention, there was already a downward trend in daily vaccination rates for both treated and control municipalities (ture 2-A). However, we see a large jump in vaccination rates on the day of the caravan visit for the treated municipalities. In Fig. 2-B we show weekly vaccination rates to avoid weekend fluctuations when almost no vaccine was administered and the pattern looks very similar.

### 4.2 Main results

**Fig. 3:**
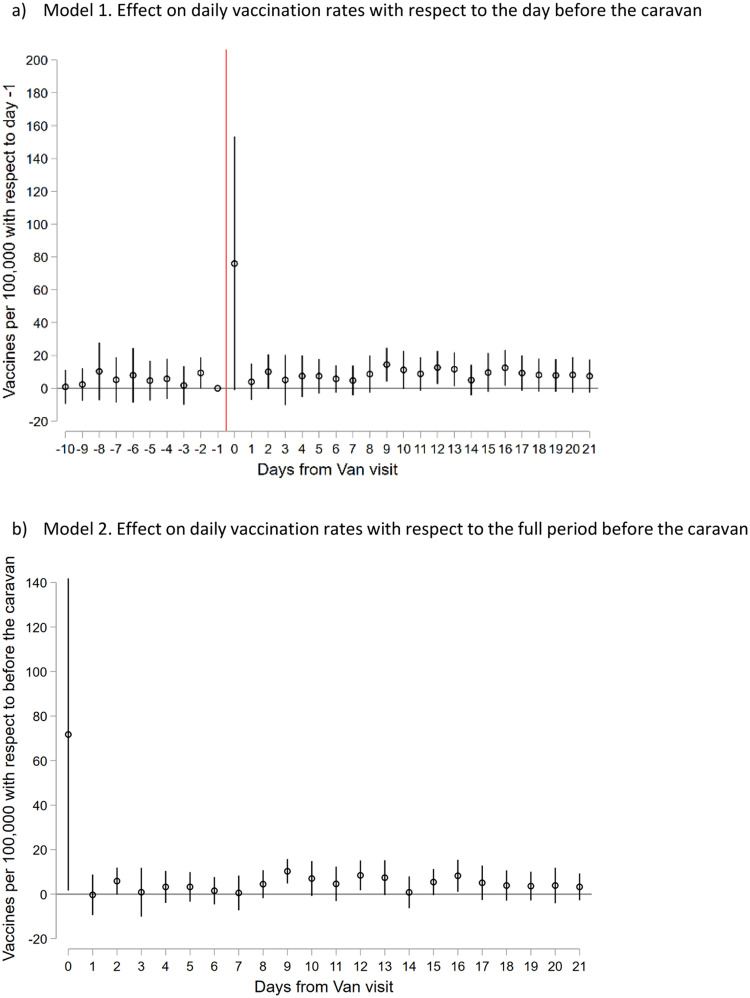
Coefficients from the event study-like DiD models 1 and 2. NOTES: Panel A reports coefficients from the “leads” (i.e.: coefficients $\gamma_{k}$ for k<0) and “lags” (i.e.: coefficients $\gamma_{k}$ for k>0) of the difference-in-difference event-study specification from model 1 (n=3,840). Panel B reports coefficients from the “lags” (i.e.: coefficients $\gamma_{k}$ for k>0) of the difference-in-difference event-study specification from model 2 (n=3,840). We have restricted the periods from -10 to +21 for graphical purposes. Coefficients for the full period are plotted in Figure A1 of Appendix A.

Fig. 3a reports the coefficient of the event study-like specification of model 1. Looking at the coefficients prior to the caravan visit we can see that the model complies with the parallel trends assumption as they are close to zero and not significant. Specifically, this shows that there are no statistically significant differences in trends prior to the intervention in treated and control municipalities. Additionally, there is a large jump in the vaccination rate of around 70 per 100,000 inhabitants on the day of the visits, a 318 % increase with respect to the pre-intervention average, suggesting a large and statistically significant immediate impact of the caravan on the day of the visit.

While the increase on the day of the visit is intuitive, and indeed if it had not been present it would have raised questions about the data, there seem to be persistent benefits for daily vaccination rates two weeks or more after the caravan visit. Importantly, both the large effect on the day of the visit and, crucially, the persisting effects are also seen in model 2 (Fig. 3b), where we compare the effect on vaccination rate with respect to the full pre-intervention period, and not only with respect to the day before the intervention.

In Table A4 of Appendix A, we report the results from the TWFE model 3. We estimate an average treatment effect on daily vaccination rates of 7.7 vaccines per 100,000 inhabitants for an average period of 3 weeks after the caravan visit. This corresponds to a 34.8 % increase with respect to pre-intervention vaccine rates.

#### 4.2.1 Heterogeneity analysis

We further carried out a heterogeneity analysis to examine whether the effect of the intervention was different according to observable municipality characteristics (e.g. population size). Specifically, we stratify the sample of municipalities based on the median value of the following characteristics (based on the North Macedonia Population Census 2021): total population, share of population aged 60 or older, share of foreign-born population, share of population with tertiary education and unemployment rate. We found no significantly different effects of the caravan by any of the characteristics examined (see Figure A2 in Appendix A). This suggests that the effectiveness of the COVID-19 mobile vaccination caravan was independent of the observable characteristics of the municipality.

### 4.3 Robustness checks

We performed the following robustness checks (Table A5 in Appendix A): i) we perform the doubly robust Callaway and Sant Anna (2020) estimation using only never-treated and not-yet treated municipalities as control municipalities. ii) we include population weights in the regression to give more weight to the results coming from larger municipalities, iii) we grouped data into weekly vaccination rates (per 1000 inhabitants) to avoid any day-specific fluctuations, and iv) we used weekly vaccination rate data jointly with population weights. Results from these robustness checks continue to show a significant increase in vaccination rates of between 24.1 % to 39.9 %, depending on the model.

### 4.4 Cost-effectiveness

**Fig. 4:**
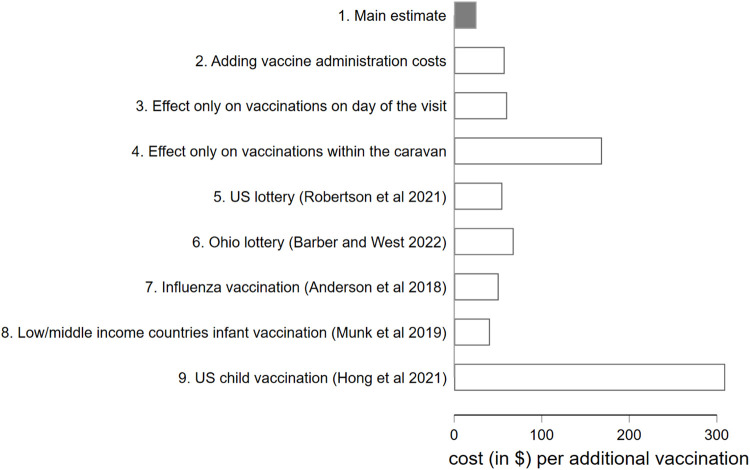
Cost-effectiveness estimations NOTES: Estimate 1 includes all vaccinations induced by the caravan up to an average of 3 weeks after the visit as estimated in the TWFE model (3) and the cost of the promotion activities. Estimate 2 is similar to Estimate 1 but adding the vaccine administration costs. Estimate 3 includes the vaccinations induced by the caravan only on the day of the visit and the cost of promotion activities. Estimate 4 includes the vaccinations administered only inside the caravan and the cost of the promotion activities. Estimate 7 corresponds to the average incremental cost per additional person vaccinated from a systematic review of interventions aimed at increasing influenza vaccine uptake [25]. Estimate 8 corresponds to the average incremental cost per additional infant vaccinated that we calculated from the estimates included in a systematic review of interventions aimed at increasing infant vaccination in low and middle-income countries [26]. Estimate 9 corresponds to the average incremental cost per additional child vaccinated that we calculated from the estimates from a systematic review of interventions aimed at increasing child vaccination in the United States (Hong et al. 2021). The cost-effectiveness calculations for the vaccine caravan are explained in detail in section D1 of Appendix D. The extraction of data from the systematic reviews for Estimates 7, 8 and 9 is explained in detail in section D2 of Appendix D.

We carried out a basic calculation of the cost per additional vaccination achieved by the intervention. Fig. 4 reports the main results (See Appendix D for detailed calculations). Our main estimate provides a cost-effectiveness estimate of the caravan of US$ 25.4 per additional vaccination, under our main scenario where we include all additional vaccinations estimated to be induced by the caravan up to 3 weeks after the visit, derived from the TWFE model, and only the cost of the vaccine promotion activities. As a benchmark, this is a cost of around half of that in vaccine lotteries in the US (US$ 55–68 per vaccination), which is, to our knowledge, the only comparable cost-effectiveness estimates of population-level interventions to promote COVID-19 vaccination available in previous literature. Systematic reviews that include interventions to encourage influenza vaccination [25], infant vaccination in low income countries [26], or child vaccination in the US (Hong et al. 2021) also report higher average cost-effectiveness estimates, as shown in Fig. 4.^5^

Even if we included the vaccine administration costs in our estimates, the cost-effectiveness would be around the same as that of the vaccine lotteries (US$ 57.6 per additional vaccine). It should be noted that if we only took into account the effect on vaccinations on *the day of the caravan visit* would have made the intervention to appear much more expensive (US$ 60.5 per additional vaccine). The intervention would look even more expensive if only vaccines administered *within the caravan* had been taken into account (US$ 168.8 per additional vaccine). This highlights the importance of implementing rigorous evaluations, by including all extra vaccinations induced by the intervention after the caravan visit (both inside the caravan and in any healthcare centre). Importantly, our cost-effectiveness estimates should be taken as a lower bound since other potential benefits are not taken into account. Among these, we could expect decreases in COVID-19 cases and hospitalizations derived both from the increase in vaccination and the dissemination of other preventive behaviours.

## 5 Discussion

We describe the effectiveness of the North Macedonia COVID-19 caravan, a community-based intervention that involved sending mobile vaccination units to different locations to increase vaccination rates. Results from our difference-in-difference analysis show that the mobile vaccination caravan had a persisting benefit by increasing vaccination rates by 35 % during the 3 weeks after the caravan visit. This implies a cost of US$ 25.4 per additional vaccination.

As with all natural experiment designs, our study has several limitations. First, we tested our analysis for ‘as-if’ randomisation across intervention and control municipalities. Although we found that they were not fully balanced on covariates, they did follow parallel trends, suggesting that areas of imbalance were not major drivers of vaccine trends and validating the main assumption of the difference-in-difference model. Second, we could not disaggregate individual recipients of vaccines to identify those who were fully vaccinated and those who were not as this information was unavailable. That said, we do know that this region has high rates of no- or incomplete-vaccination status. Third, we observed a spike in vaccinations on the day of the caravan, as well as an enduring impact over time. While we cannot fully disentangle the mechanisms underlying this association, a recent systematic review has shown how a wide range of “nudge” interventions can increase vaccine uptake although their impact is highly context-dependent [27]. In this case, increased uptake after the visit could plausibly be explained by i) saliency effect – through promotional activities in the lead-up to the caravan by community leaders and international partners, which may have influenced social norms, and ii) dissemination effect – arising from the diffusion of public health advice from word-of-mouth from family, friends and other peer networks.

Our study corroborates evidence from prior studies of mobile units conducted in other settings. One, in the UK, produced results similar in magnitude (23 % increase in vaccination) [13], and another in Switzerland showed a very large increase of more than threefold in vaccination [14]. In Pakistan, a LMIC, a similar intervention increased vaccination rates by 17 % [18]. Our analysis validates the effectiveness of these measures, including their cost-effectiveness, in a higher middle-income country with a vaccine-hesitant environment.

Arguably the most important finding in our study was the persisting effect of the caravan on vaccine uptake. When considering the vaccine uptake solely attributable to the day of the caravan visit, our cost-effectiveness estimates indicated a cost of US$ 60.5 per additional vaccine, increasing up to US$ 168.80 if we only included vaccine administered within the caravan. However, when we included the enduring effect observed in the three weeks following the visit, including vaccines obtained both within the caravan and at other healthcare facility, the cost-effectiveness significantly improved to US$ 25.40 per additional vaccine. This suggests that the promotion activities may have played an important role in the overall effectiveness of the vaccine caravan. Additionally, it underscores the importance of considering lagged effects in community-based interventions, even when the intervention itself has a short duration. Including such temporal effects can have a substantial impact on the overall estimated effectiveness of the intervention.

## 6 Conclusions

Taken together, our results show that mobile vaccination units can be an effective tool to increase COVID-19 vaccination rates, even in the context of persistent suboptimal vaccine uptake. It demonstrates cost-effectiveness in comparison to alternative approaches, such as lotteries. This may provide useful evidence for policymakers who are struggling to promote vaccination within countries where most of the population is still unvaccinated.

## CRediT authorship contribution statement

**Manuel Serrano-Alarcón:** Conceptualization, Data curation, Formal analysis, Methodology, Writing – original draft. **Martin Mckee:** Conceptualization, Writing – review & editing. **Leonardo Palumbo:** Conceptualization, Writing – review & editing. **Cristiana Salvi:** Conceptualization, Writing – review & editing. **Anne Johansen:** Writing – review & editing. **David Stuckler:** Conceptualization, Writing – review & editing.

## Declaration of Competing Interest

Authors declare no conflict of interest. The researchers have carried out the analysis independently of the funders of the intervention. WHO Europe only provided the data while it had no role on the design of the study, methodological choices, data analysis nor on the presentation and interpretation of the results.

## Appendix Supplementary materials

Image, application 1

## Footnotes

1 The share of the population varied by age group: 67.7% for those aged 50 and older, 51.9% for those aged 25 to 49, 38.60% for those aged 18 to 24 and only 1% for those younger than 18 years old. (Data provided by WHO Europe as of March 2022)

2 As explained in section 2, the work of the vaccine caravan extended beyond providing on-site vaccination. It also included vaccine promotion activities, encompassing one to one discussions with health workers, media dissemination and the distribution of informational brochures. One may expect that these activities incentivize vaccination uptake not only within the caravan but also in other local healthcare facilities both the day of the caravan visit and on subsequent dates. For more details on the promotion activities of the caravan, please refer to Appendix B.

3 We utilise data up to 17^th^ of April. As the intervention was staggered from 21th of March to 3^rd^ April, we observe “treated” municipalities between a maximum of four weeks (if they received the caravan on 21th of March) and a minimum of two weeks (if they received the caravan on 3^rd^ of April). As a consequence, “treated” municipalities were observed on average up to three weeks after the visit. This is why we report our main results up to day 21 after the caravan visit. The full period results can be found in Appendix A.

4 The main advantage of model 1 is that it can simultaneously test for the presence of pre-treatment parallel trends and for any post-intervention lagged effects. However, these estimates can be biased by the existence of any anticipation effects (i.e. if individuals change their vaccination behaviour before the intervention due to the announcement of the caravan visit). On the other hand, model 2 avoids any anticipation effects as its reference category is formed by the whole period before the intervention. As shown in section 4.2 both models show very similar results, suggesting that there anticipation effects are not present.

5 There is, however, much variation in the cost-effectiveness estimates of interventions to promote vaccination in the previous literature. For instance, estimates from interventions aimed to increase influenza vaccination uptake ranged from US$ 6.26 to US$ 854.07 per additional vaccination [25].
