# Supplementary material for: How to increase COVID-19 vaccination among a population with persistently suboptimal vaccine uptake? Evidence from the North Macedonia mobile vaccination and public health advice caravan
Source: Health Policy. 2024 Jan;139:104966. doi: 10.1016/j.healthpol.2023.104966 (PMC10788482; doi:10.1016/j.healthpol.2023.104966)
Supplement: Supplementary file 1 [file mmc1.docx]

**Appendix A. Additional results**

Table A1 – Timing of the caravan visit per treated municipality

| **Date** | **Municipality of the location** | **Treated municipalities** |
| --- | --- | --- |
| 3/21/2022 | Skopje (Suto Orizari) | Skopje (Suto Orizari) |
| 3/22/2022 | Kratovo | Kratovo |
| 3/23/2022 | Radovis | Radovis |
| 3/24/2022 | Strumica | Strumica |
| 3/25/2022 | Novo Selo | Novo Selo |
| 3/26/2022 | Skopje (Gazi Baba) | Skopje (Gazi Baba), Aerodrom Skopje, Butel Skopje, Centar Skopje, Chair Skopje, Karposh Skopje, Kisela Voda Skopje, Saraj Skopje |
| 3/27/2022 | Kumanovo | Kumanovo |
| 3/28/2022 | Lipkovo | Lipkovo |
| 3/29/2022 | Aracinovo | Aracinovo |
| 3/30/2022 | Cucer Sandevo | Cucer Sandevo |
| 3/31/2022 | Zelenikovo | Zelenikovo |
| 4/1/2022 | Vrapciste | Vrapciste |
| 4/2/2022 | Kicevo | Kicevo |
| 4/3/2022 | Struga | Struga |

Table A2 – Goodman-Bacon decomposition results

| DD Comparison | Weight | Average DD estimate |
| --- | --- | --- |
| Earlier Treated vs Later Treated | 0.014 | 20.04 |
| Later Treated vs Earlier treated | 0.023 | 25.41 |
| Treated vs Never treated | 0.962 | 5.68 |

NOTE: Results from the Goodman-Bacon (2021) decomposition of the difference in difference (DD) performed using the “ddtiming” STATA command.

Table A3 – Difference in means between treated and control municipalities

|  | (1) Treated | | (2) Control | | Difference in means^a^ |
| --- | --- | --- | --- | --- | --- |
|  | Mean | SD | Mean | SD | (1) - (2) |
|  |  |  |  |  |  |
| Population | 39603.5 | 25685.2 | 16645.5 | 19641.1 | 22957.989*** |
| Age 0-9 | 0.119 | 0.031 | 0.102 | 0.026 | 0.017* |
| Age 10-19 | 0.119 | 0.027 | 0.111 | 0.02 | 0.008 |
| Age 20-29 | 0.126 | 0.025 | 0.118 | 0.015 | 0.008 |
| Age 30-39 | 0.134 | 0.014 | 0.129 | 0.011 | 0.004 |
| Age 40-49 | 0.137 | 0.011 | 0.134 | 0.009 | 0.002 |
| Age 50-59 | 0.136 | 0.016 | 0.146 | 0.014 | -0.010* |
| Age 60-69 | 0.124 | 0.032 | 0.142 | 0.026 | -0.018* |
| Age 70-79 | 0.076 | 0.026 | 0.084 | 0.019 | -0.007 |
| Age 80+ | 0.03 | 0.013 | 0.033 | 0.01 | -0.004 |
| No education completed | 0.051 | 0.047 | 0.075 | 0.05 | -0.024 |
| Primary education | 0.243 | 0.124 | 0.283 | 0.085 | -0.04 |
| Secondary education | 0.346 | 0.097 | 0.357 | 0.097 | -0.011 |
| Tertiary education | 0.154 | 0.113 | 0.104 | 0.052 | 0.051 |
| Foreign-born | 0.029 | 0.016 | 0.021 | 0.015 | 0.008* |
| Ethnicity: North Macedonia | 0.51 | 0.339 | 0.672 | 0.306 | -0.162 |
| Ethnicity: Albanian | 0.307 | 0.344 | 0.142 | 0.26 | 0.165 |
| Ethnicity: other | 0.183 | 0.122 | 0.186 | 0.181 | -0.002 |
| Unemployment rate | 0.229 | 0.203 | 0.217 | 0.146 | 0.012 |
| N | 22 |  | 58 |  | 80 |

NOTES: ^a^ p-values of the independent sample t-test for the difference in means: *** p<0.01, ** p<0.05, * p<0.1

Figure A1 - Coefficients from the event study-like DiD models 1 and 2. Full period

| 1. Model 1. Effect on daily vaccination rates with respect to day before the caravan |
| --- |
| 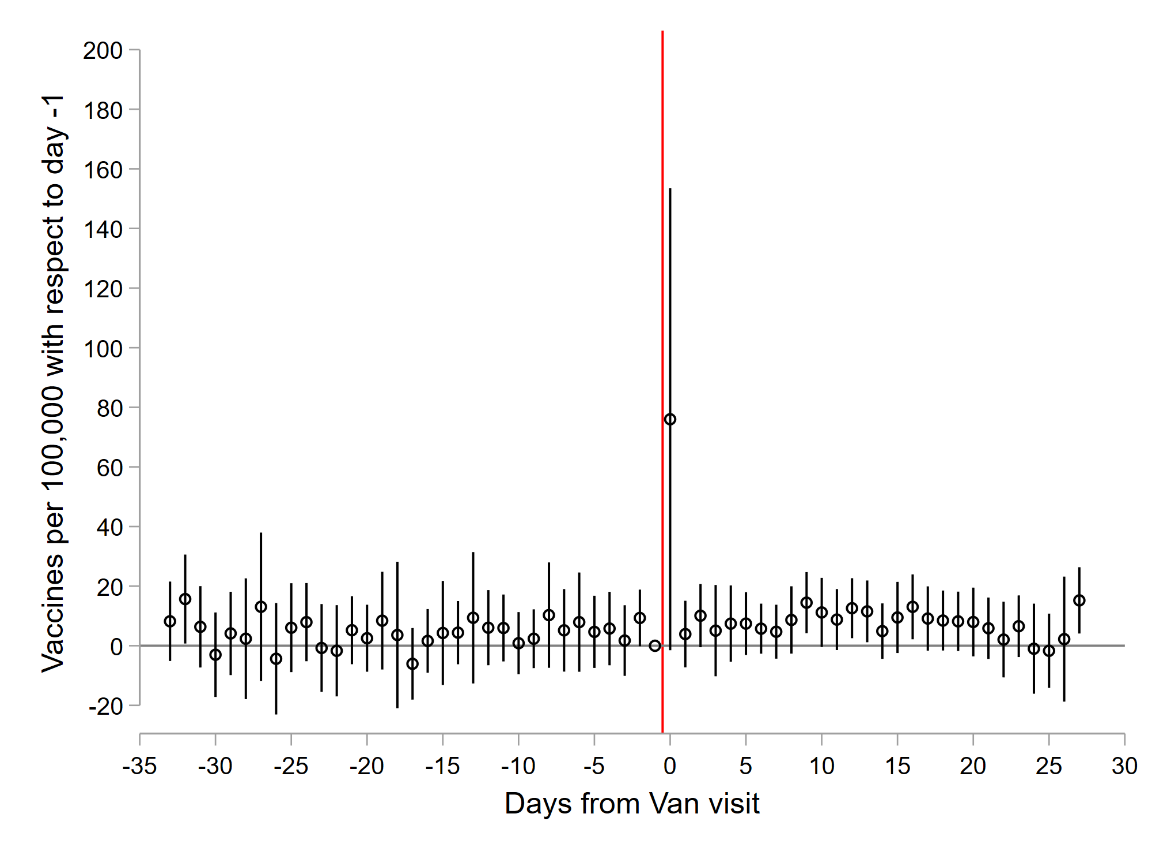 |
| 1. Model 2. Effect on daily vaccination rates with respect to the full period before the caravan |
| 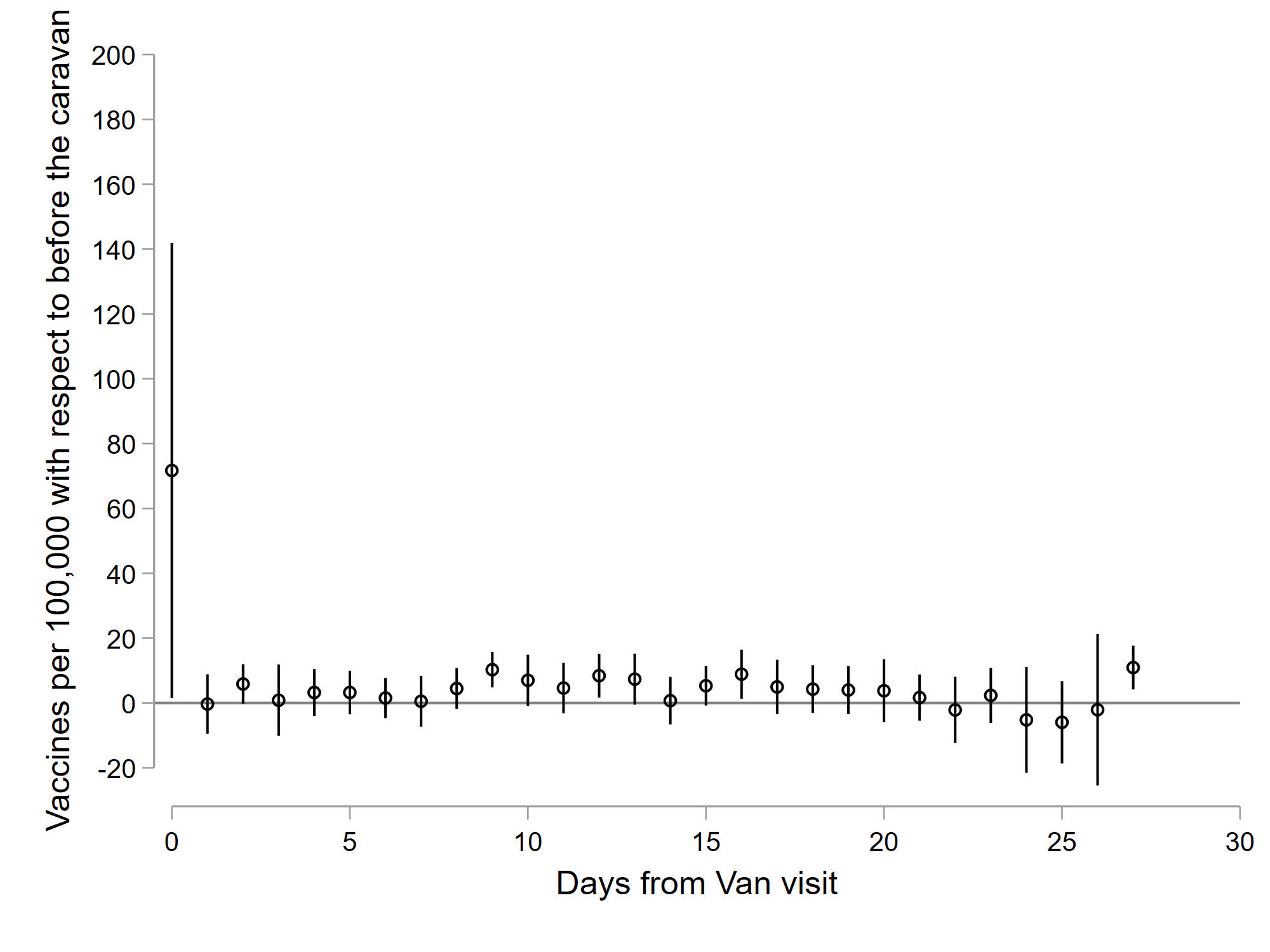 |

NOTES: Panel A reports coefficients from the “leads” (i.e.: coefficients $\gamma_{k}$ for k<0) and “lags” (i.e.: coefficients $\gamma_{k}$ for k>0) of the difference-in-difference event-study specification from model 1 (n=3,840). Panel B reports coefficients from the “lags” (i.e.: coefficients $\gamma_{k}$ for k>0) of the difference-in-difference event-study specification from model 2 (n=3,840).

Table A4- Result from TWFE model. Average treatment effect of the intervention on daily vaccine rates

|  | (1) |
| --- | --- |
|  | TWFE |
|  |  |
| Effect on vaccine rate$(\beta_{1}Caravan$) | **7.771**** |
|  | **(3.791)** |
|  |  |
| Observations | 3,840 |
| Mean Y before treatment for the treated | 22.28 |
| Relative increase with respect to the mean | 34.88% |

NOTES: Standard errors cluster at municipality in level in parenthesis *** p<0.01, ** p<0.05, * p<0.1

Figure A3 - TWFE results by characteristics of the population of the municipalities.


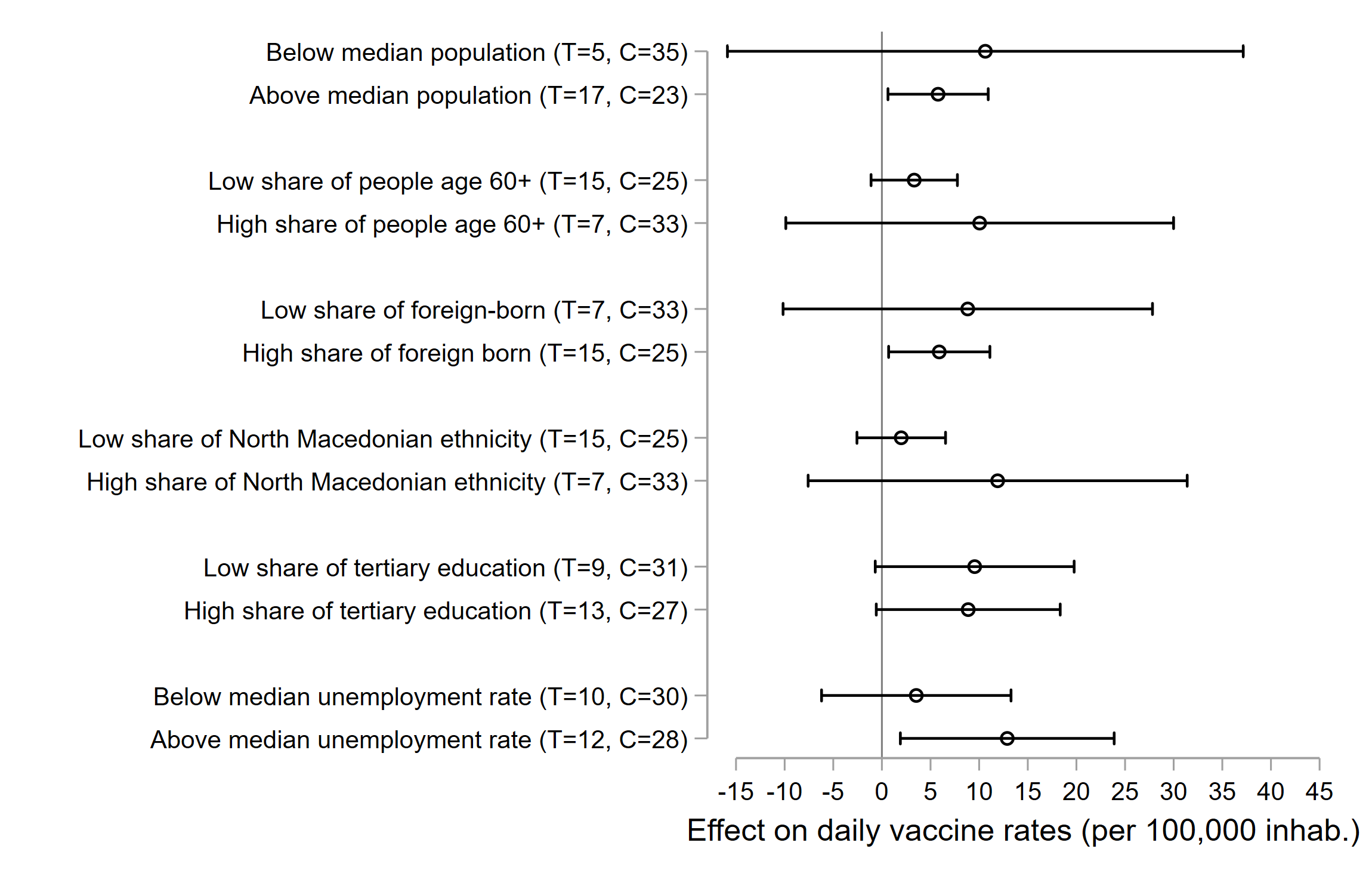


NOTES: Each coefficient (and 95% confidence interval) reports the effect of the COVID-19 caravan from the TWFE regression (equation 2 in main text) derived from a subsample. By order, we create the subsamples by splitting the sample of municipalities based on the median value of: total population, share of population age 60 or more, share of population foreign-born, share of population with North Macedonian ethnicity, share of population with tertiary education completed, unemployment rate. Data from these variables comes from the North Macedonia Population Census 2021 (Source: MAKStat Database https://makstat.stat.gov.mk/PXWeb/pxweb/en/MakStat/?rxid=46ee0f64-2992-4b45-a2d9-cb4e5f7ec5ef) T-number of treated municipalities, C-number of control municipalities.

Table A5- Robustness checks for the TWFE model

|  | (1) | (2) |  | (3) | (4) |
| --- | --- | --- | --- | --- | --- |
| VARIABLES | Sant Anna and Zhao (2020) | Population weights |  | Weekly vaccine rate | Weekly vaccine rate & population weights |
| TWFE effect on vaccine rate $(\beta_{1}Caravan$) | 8.903** | 4.554** |  | 46.08* | 27.28** |
|  | (4.327) | (1.945) |  | (24.611) | (12.932) |
|  |  |  |  |  |  |
| Observations | 3,840 | 3,840 |  | 560 | 560 |
| Mean Y before treatment for the treated | 22.28 | 16.89 |  | 161.9 | 113.2 |
| Relative increase with respect to the mean | 39.96% | 26.96% |  | 28.47% | 24.12% |

NOTES: Column (1) reports the results from the Sant’Anna and Zhao (2020) doubly robust difference in difference estimator based on stabilized inverse probability weighting and ordinary least squares using as dependent variable the daily vaccine rate per 100,000 inhabitants (“csdid” command in Stata). Column (2) reports the results from the two way fixed effects model including population weights and using as dependent variable the daily vaccine rate per 100,000 inhabitants. Column (3) reports the results from the two way fixed effects using as dependent variable the weekly vaccine rates per 100,000 inhabitants. Column (4) reports the results from the two-way fixed effects including population weights and using as dependent variable the weekly vaccine

**Appendix B – Implementation of joint MoH/WHO/UNICEF/USAIDCOVID-19 vaccination caravan in North Macedonia**

**Background:**

Under the USAID-funded project for immunization, WHO will partner with the Ministry of Health to develop and organize community-based outreach activities for raising awareness for COVID-19 vaccination and protective measures, through implementation of information and vaccination caravans in areas with low COVID-19 vaccination uptake, in collaboration with the national health authorities. These community level interventions will highlight the benefits from immunization and encourage active involvement of all relevant actors from the community (e.g. community leaders, religious leaders, NGOs, Mayors etc.) who can contribute to change people's perception on COVID-19 vaccination, taking into account the cultural, religious and demographic features of the targeted communities.

Furthermore, this activity will help to further explore community engagement and enabling interventions wherein community members play a significant role in enhancing COVID-19 vaccination. Effective communication on the importance of vaccination, raising public awareness on the importance and significance of the COVID-19 immunization process and bringing vaccination closer to communities will contribute toward increasing COVID-19 vaccination uptake.

**Objectives:**

- Encourage COVID-19 vaccination uptake among the general public and promote acceptance of preventive behaviours and public health measures;
- Ensure that COVID-19 vaccination is easily accessible in local communities, especially for vulnerable population and at-risk communities such as the elderly;
- Promote participation of community leaders in local outreach activities to work out barriers to the uptake of COVID-19 vaccination and public health measures.

**Visual identity and branding**

The overall slogan for the caravan will be “I got vaccinated – vaccines save lives”.

*Key visual identity:*


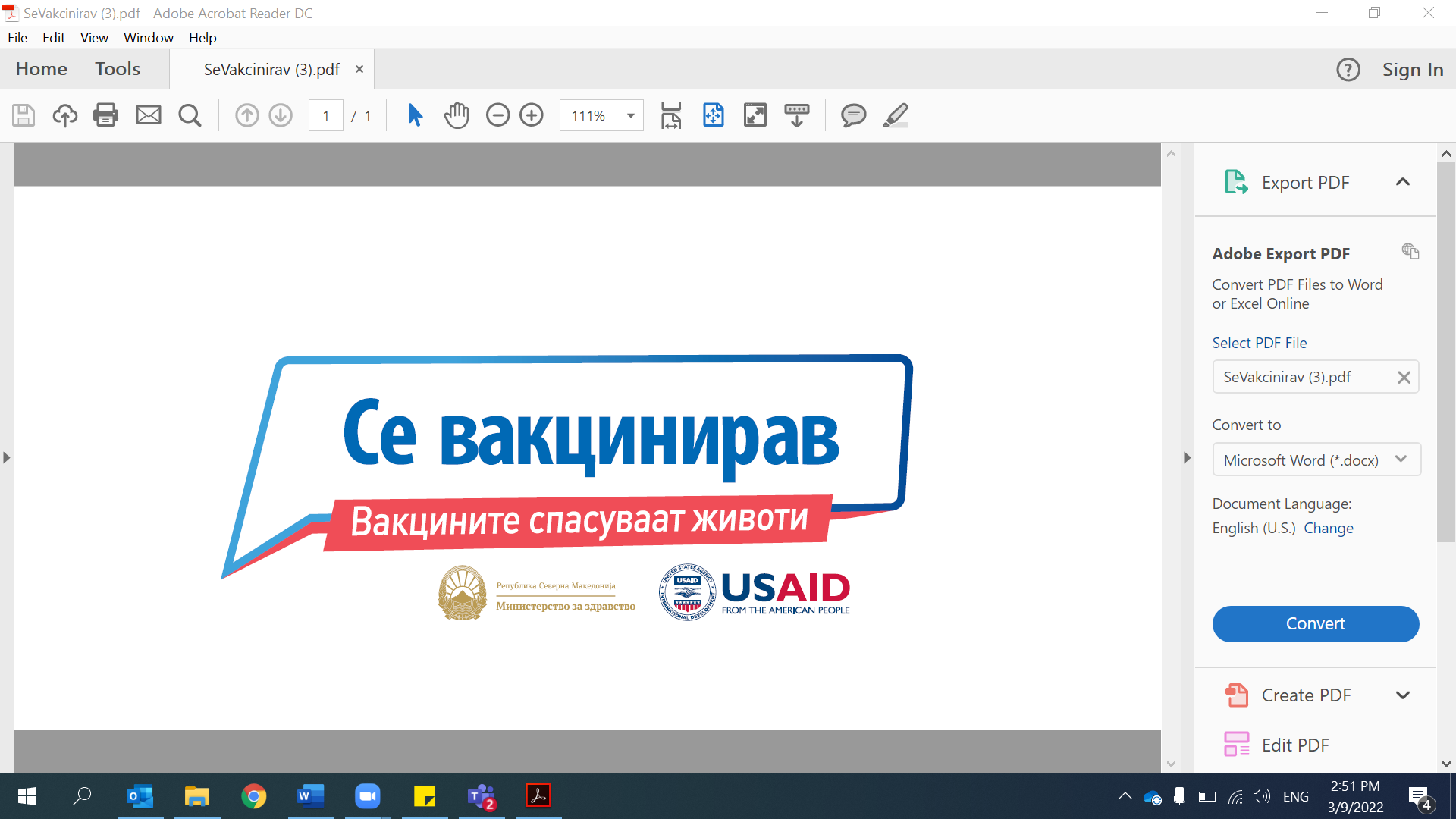


**Event setup**

WHO CO will support the logistical setup of each event, including include:

- Branded banners and backdrops (in local languages)
- Tent – 12m x 6m;
- Info corner (6m x 3m);
- Vaccination corner (6m x 3m); and
- Waiting/rest corner (6m x 6m).

WHO CO will organize all logistical aspects for the events setup, while the MoH will organize all aspects of the COVID-19 vaccination process, including organizing health care teams for the events, vaccines and other medical supplies and equipment as needed.

*Preview of the event setup:*


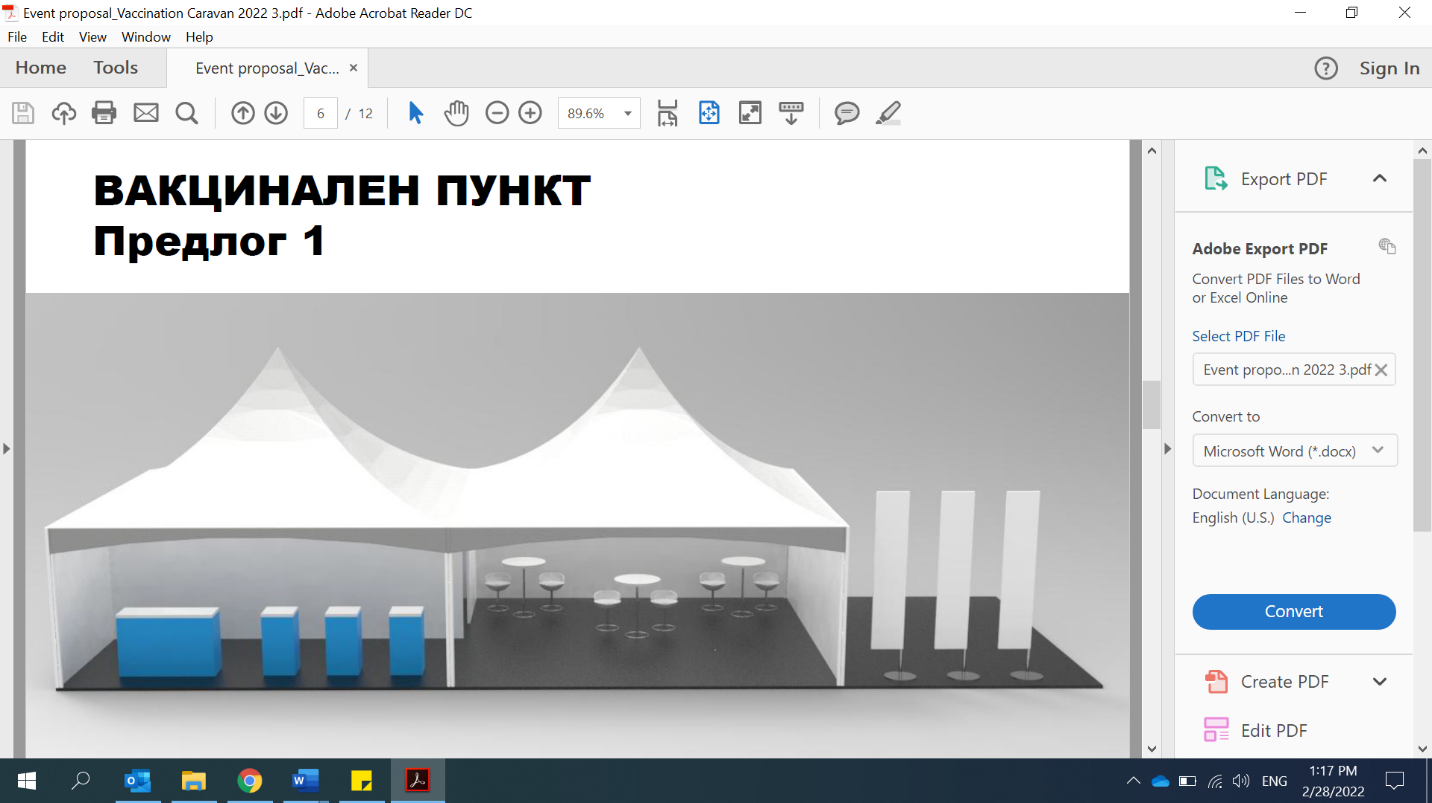


.


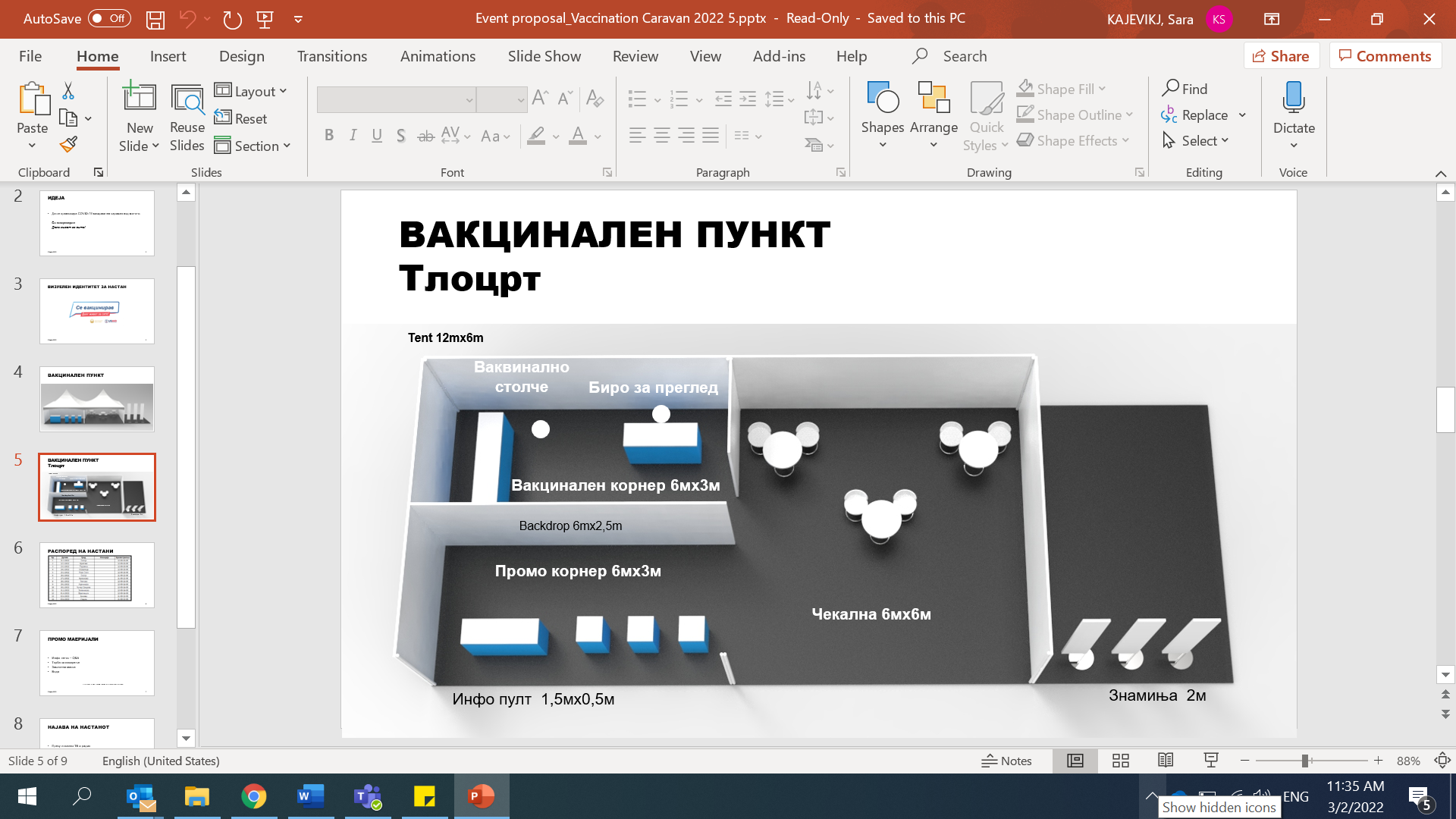


**Promotional and informational materials**

A set of branded promotional/informational materials will be disseminated during the events, to provide accurate and up to date information on COVID-19 vaccination and encourage uptake of vaccination as well as preventive measures and behaviors.

Branded gift bags will be disseminated to visitors, along with:

- Brochure with Q&A on COVID-19 vaccination (in local languages);
- Hand sanitizers and masks; and
- Water.

**Outreach activities**

| **Activity** | **Details** | **Responsible partner** |
| --- | --- | --- |
| Develop promotional materials | Based on the key visual, develop social media tiles for each separate location into local languages as needed  Adapt social media tiles into posters and flyers as needed (format)  Develop message kit to be used in press releases, social media, talking points etc. | Design – UNICEF, with WHO support as needed |
| Prepare draft press release to be used by MoH and municipalities | MoH to share the press release with all municipalities   Municipalities to disseminate press releases prior to the events | MoH, with support from UNICEF and WHO to prepare the draft |
| Organize TV appearances into local media outlets | Arrange guest appearances of local health professionals to share information on COVID-19 and announce the events | MoH |
| Social media outreach | Promote the tiles on social media platforms and create events as needed | UNICEF |
| Dissemination of flyers/posters | Organize dissemination of materials before the event in cooperation with CSOs | WHO |
| Outreach to Moj Termin | Sending SMS notification to citizens per region 24 hours prior the event  Create 160 characters draft text message that will be sent to the citizens | WHO, MoH |
| Outreach through municipalities | Inform all municipalities about the upcoming caravan and encourage them to disseminate information through local media etc. | MoH |
| Health care workers | Share information with all family doctors through the Association and encourage them to inform patients   Share information with Doctors Chamber to disseminate the information to all doctors and partners  Share information through the Nurses Association   Share information with HCWs from the local health centers | WHO, MoH |
| Religious leaders | Disseminate information to religious leaders and encourage them to further spread information | MoH |
| Photo gallery and video post-caravan |  | WHO |

**Appendix C – Methodological Appendix**

We exploit the staggered implementation of the COVID-19 vaccine caravan by comparing the evolution of vaccine rates among those municipalities who received a visit of the caravan vs those who did not in a series of difference-in-difference (DiD) models. In total, 22 municipalities received the caravan and 58 did not.

First, we utilize a DiD model in an event study-like specification to study the effect of the intervention relative to the time of the caravan visit, as follows:

$y_{m,t}=\beta_{0}+ \sum_{-k}^{K} \gamma_{k}{Caravan visit for k periods}_{i,t}+\delta_{t}+\alpha_{m}+ \varepsilon_{i,t}$ (1)

Where $y_{mt}$ is the vaccine rate per 100,000 inhabitants. We include time fixed effects $(\delta_{t})$ and municipality fixed effects ($\alpha_{m}$). k=0 is the day of the caravan visit. Coefficients $\gamma_{k}$ for k<0 (“leads”) test for the presence of pre-treatment parallel trends, a key assumption in the difference-in-difference model as it is necessary to show that there were no differences in trends in treated and control municipalities before the intervention. For this to be true the coefficients prior to the intervention (k<0) should not be statistically significant from zero. Coefficients $\gamma_{k}$ for k>0 (“lags”) measure the effect of the caravan visit k days after, with respect to the day before the caravan visit, which we set as the baseline in our model (k=-1).

Second, in order to study the dynamics of the effect over time further and avoid setting an arbitrary day as baseline for our estimates, we estimate the following model excluding the “leads”, as follows.

$y_{m,t}=\beta_{0}+ \sum_{k=0}^{K} \gamma_{k}{Caravan visit for k periods}_{i,t}+\delta_{t}+\alpha_{m}+ \varepsilon_{i,t}$ (2)

In model (2), coefficients $\gamma_{k}$ for k>0 measure the effect of the caravan visit k days after, with respect to the full period before the caravan visit, not only with respect to the day before (as it was in model 1). The rest of the variables are similar to those included in model (1).

Finally, we calculate the average treatment effect for the full post-treatment period with the following two-way fixed effects (TWFE) model:

$y_{m,t}= \beta_{0}+{\beta_{1}Caravan}_{m,t}+\delta_{t}+\alpha_{m}+ \varepsilon_{i,t}$ (3)

Where ${Caravan}_{m,t}$ equals one if the caravan has already visited the municipality m by time t, and zero otherwise. $\beta_{1}$ measures the average treatment effect on daily vaccine rates during the three weeks after the intervention. The rest of the variables are similar to those included in models (1) and (2).

**Appendix D. Costs and cost-effectiveness calculation**

**D.1. Cost and cost-effectiveness calculations of the vaccine caravan**

This section provides the results of our back-of-the-envelope cost-effectiveness calculation of the COVID-19 vaccine caravan. First, in table D1 we summarize the effectiveness results. Briefly, in our main analysis (column a) using the TWFE model we estimated an increase in vaccination rates of 7.77 per 100,000 inhabitants per day during an average period of 3 weeks following the caravan visit. Multiplying this by the average population in the treated municipality (39,603) results in an increase of 3.08 vaccines per municipality per day. This equals to an increase of 67.71 per municipality during our period of analysis (up to 17^th^ of April)^[[1]](#footnote-1)^. Since we have 22 treated municipalities, in total we estimate that there were an additional 1,489 vaccines injected due to the caravan visit (67.71 x 22= 1,489). In Table D2, we report a summary of the costs of the caravan. The cost of the promotion activities was 37,808 USD whereas the total cost of the whole intervention (including vaccine administration costs) was 85,841 USD. In Table D3, we report the cost-effectiveness estimations. In our main analysis, we take into consideration only the cost of the vaccine promotion activities as we are estimating the cost-effectiveness of an additional vaccine *induced* by the caravan. The administration costs would always be needed in the alternative scenario of vaccination without the caravan. Under this assumption, we estimate a cost-effectiveness of 25.4 USD per additional vaccination (Table D3, column a, first row). For comparison purposes we provide the effectiveness (Table D1) and cost-effectiveness (Table D3) estimations of two other alternative scenarios: taking into account only the effect of the caravan on the day of the visit (column b: 60.5 USD per additional vaccination) and taking into account only vaccinations administered inside the caravan (column c: 168.8 USD per additional vaccination).

Table D1 - Effectiveness summary

|  | a) Effect on the full period (up to 3 weeks after caravan visit). Main results from TWFE model | b) Effect only on the day of the caravan visit | c) Effect on the day of the caravan visit. Only vaccinations in-caravan |
| --- | --- | --- | --- |
| Effect on daily vaccination rate (per 100,000) | 7.77 | 71.72 |  |
| Effect on daily vaccines per municipality ^a^ | 3.08 | 28.40 |  |
| Effect on total daily vaccines ^b^ | 67.71 | 624.87 |  |
| **Effect on total vaccines over the period** | **1489.53** ^c^ | **624.87** | **224** ^d^ |

NOTES: ^a^ Effect on daily vaccines per municipality = effect on daily vaccinate rate (per 100,000) x 39,603 (average population in the treated municipality)/100,000. ^b^ Effect on total daily vaccines = daily vaccines per municipality x 22 (number of treated municipalities). ^c^ On average, we observe each treated municipality for 22 days after the caravan visit as of 17^th^ of April, our last day of observation (67.71 total daily vaccines x 22 days = 1489.53 total vaccines over the period). ^d^ Data provided by WHO Europe.

Table D2- Costs summary

|  | **Total** |  |
| --- | --- | --- |
|  | MKD | USD |
| **(1) Costs of vaccine promotion activities** | **2,109,630** | **37,808** |
| (2) Cost of vaccine administration | 2,680,200 | 48,033 |
| Total costs (1) + (2) | 4,789,830 | 85,841 |

Note: We used the exchanged rate as of 21^st^ of March (1 MKD = 0.0179215 USD)

Table D3 - Cost effectiveness summary

|  | a) Effect on the full period (up to 3 weeks after caravan visit). Main results from TWFE model | | b) Effect only on the day of the caravan visit | | | c) Effect on the day of the caravan visit. Only vaccinations administered in-caravan | | | |
| --- | --- | --- | --- | --- | --- | --- | --- | --- | --- |
| Cost per additional vaccine | MKD | USD | MKD | USD | | MKD | USD | |  |
| Including only costs of promotion activities (1) | 1416.3 | **25.4** | 3376.1 | | **60.5** | 9418.0 | **168.8** |  |  |
| Including all costs (1 + 2) | 3215.7 | **57.6** | 7665.3 | | 137.4 | 21383.2 | 383.2 |  |  |

**D.2. Cost-effectiveness estimates from related literature**.

In this section we provide information on how we extracted the cost-effectiveness data from previous studies that reported the cost-effectiveness of interventions that aimed to increase vaccine uptake of other diseases. In particular, we extract data from the following three systematic reviews:

*Anderson et al (2018). The cost of interventions to increase influenza vaccination: a systematic review. American journal of preventive medicine, 54(2), 299-315.*

This systematic review included papers published from January 2004 to July 2016 which evaluated the cost-effectiveness of interventions by bodies within the healthcare system to increase influenza vaccination by means of an organizational or structural change. In particular, it focused on healthcare-based quality improvement (QI) interventions. These interventions included, among others, physician reminders, standing order programs, patient reminders, pharmacist-lead vaccine programs, counselling services in physician practices or reduction of out of pocket costs and co-payments. The review included 29 interventions. 22 were based in the U.S., three in the United Kingdom, two in Japan, one in Switzerland, and one in Germany. Estimates varied from US$ 6.26 to US$ 854.07 per additional person vaccinated. The study reports **an average incremental cost per additional person vaccinated of US$ 50.78** derived from the 23 interventions in the general population included in the review.

*Munk et al (2019). Systematic review of the costs and effectiveness of interventions to increase infant vaccination coverage in low-and middle-income countries. BMC health services research, 19(1), 1-10.*

This systematic review included papers published from January 2003 to May 2019 which evaluated the cost-effectiveness of interventions designed to increase infant vaccination coverage in low- and middle-income countries. These interventions included demand generation, modified delivery approaches, cash transfer programs, health systems strengthening, and novel technology usage. They identified 14 studies. 10 estimates came from Asia, with the remaining coming from Africa (Ethiopia, Guinea-Bissau, and Madagascar) and Central America (Mexico and Nicaragua). Average incremental cost per additional vaccinated infant was calculated for both studies which included a control group and studies with only a pre-post comparison without control group. Estimates varied from US$ 0.66 to US$ 161.95 (Table D4). However, they do not provide an average incremental cost for the sample of interventions included in the review. We calculate a simple average of the estimates reported in the systematic review in Table D4 below. This figure shows an average **incremental cost per additional infant vaccinated of US$ 40.85**

*Hong et al (2021). Costs of interventions to increase vaccination coverage among children in the United States: a systematic review. Academic pediatrics, 21(4), S67-S77.*

This systematic review included papers published from January 2009 to August 2019 which evaluated the cost-effectiveness of interventions designed to increase vaccination coverage among children and adolescents in the United States. They identified 37 studies. Average incremental cost per additional vaccinated child was calculated only for studies which included a control group. The review reported the average incremental cost per additional vaccinated child by type of intervention. Estimates vary from an average of US$ 25.67 for a healthcare system-based combined intervention with multiple components to US$ 5,098.57 for a community-based combined intervention with multiple components (Table D5). However, they do not provide an average incremental cost for the full sample of interventions, nor for each single intervention. In order to provide a comparable summary incremental cost we calculate an average incremental cost weighted by the number of estimates included in each type of intervention group. These calculations are summarized in Table D5 below. This figure shows an average **incremental cost per additional child vaccinated of US$ 309.6**

Table D4 – Estimates of Incremental cost per infant vaccinated from Munk et al (2019)

| **Study** | **Intervention/estimate** | **Incremental cost per additional infant vaccinated (USD 2017)** |
| --- | --- | --- |
| Andersson 2009 | Measles | 124.86 |
|  | DPT3 | 119.43 |
| Banerjee 2010 | Intervention A | 1.09 |
|  | Intervention B | 0.66 |
| Byberg 2017 |  | 3.29 |
| Drain 2003 | Auto-disable syringes | 78.06 |
|  | Mixed syringes | 5.03 |
| Hayford 2014 |  | 41.4 |
| Khan 2013 |  | 5.5 |
| Levin 2005 |  | 1 |
| Pandey 2007 |  | 6.88 |
| Powell-Jackson 2018 |  | 161.95 |
| Rainey 2009 |  | 9.01 |
| Soeung 2006 |  | 13.75 |
|  |  |  |
| **Simple average** |  | **US$ 40.85** |

NOTES: This information has been extracted from Table 2 of Munk et al (2019). We only include estimates and studies for which the author were able to calculate the incremental cost per additional infant vaccinated

Table D5 - Estimates of Incremental cost per child vaccinated from Hong et al (2021)

|  | **Incremental Cost per additional child vaccinated** |  |
| --- | --- | --- |
| **Type of intervention** | **Sample-weighted mean (2019 USD)** | **Number of estimates/measures** |
| Enhancing access to vaccination services. Vaccination programs in schools (all age groups) | 82.72 | 5 |
| Client reminder and recall systems (all age groups) | 173.74 | 18 |
| Provider assessment and feedback (11−17 y old) | 63.29 | 1 |
| Community-based (0−10 y old): reminder/recall, case management and home visit# | 5098.57 | 1 |
| Community-based (11−17 y old): patient tracking, reminder/recall, and home visit | 767.02 | 1 |
| Healthcare system-based (11−17 y old): education, electronic alerts, audit and feedback, and reminder/recall | 25.67 | 5 |
|  |  |  |
| **Average (**weighted by number of estimates in each type of intervention group**)** | **US$ 309.62** |  |

NOTES: This information has been extracted from Table 1 of Hong et al (2021). We only include type of intervention groups for which the author were able to calculate the average incremental cost per additional child vaccinated.

1. On average, we observe each treated municipality for 22 days after the caravan visit as of 17^th^ of April, our last day of observation (3.08 vaccines per municipality per day x 22 days = 67.71 vaccines per municipality). [↑](#footnote-ref-1)
